# Naringin and naringenin counteract taxol-induced liver injury in Wistar rats via suppression of oxidative stress, apoptosis and inflammation

**DOI:** 10.1007/s11356-023-28454-4

**Published:** 2023-07-19

**Authors:** Shimaa S. Khaled, Hanan A. Soliman, Mohammed Abdel-Gabbar, Noha A. Ahmed, El-Shaymaa El-Nahass, Osama M. Ahmed

**Affiliations:** 1 Biochemistry Department, Faculty of Science, Beni-Suef University, P.O. Box 62521, Beni-Suef, Egypt; 2 Physiology Division, Zoology Department, Faculty of Science, Beni-Suef University, P.O. Box 62521, Beni-Suef, Egypt; 3 Department of Pathology, Faculty of Veterinary Medicine, Beni-Suef University, P.O. Box 62521, Beni-Suef, Egypt

**Keywords:** Naringin, Naringenin, Taxol, Hepatoprotective, Oxidative stress, Apoptosis

## Abstract

This research aimed to evaluate the preventing effects of naringin, naringenin, and their combination on liver injury induced by Taxol (paclitaxel) in Wistar rats. Male Wistar rats received 2 mg/kg Taxol intraperitoneal injections twice weekly on the second and fifth days of each week for 6 weeks. During the same period as Taxol administration, rats were given naringin, naringenin, or a combination of the two (10 mg/kg b.wt) every other day. Treatment with naringin and/or naringenin reduced the abnormally high serum levels of total bilirubin, aspartate transaminase, alanine transaminase, alkaline phosphatase, lactate dehydrogenase, and gamma-glutamyl transferase in Taxol-treated rats. It also significantly increased the level of serum albumin, indicating an improvement in the liver. The perturbed histological liver changes were markedly improved due to the naringin and/or naringenin treatment in Taxol-administered rats. Additionally, the treatments reduced high hepatic lipid peroxidation and increased liver glutathione content as well as the activities of superoxide dismutase and glutathione peroxidase. Furthermore, the treatments reduced the levels of alpha-fetoprotein and caspase-3, a pro-apoptotic mediator. The naringin and naringenin mixture appeared more effective in improving organ function and structural integrity. In conclusion, naringin and naringenin are suggested to employ their hepatoprotective benefits via boosting the body’s antioxidant defense system, reducing inflammation, and suppressing apoptosis.

**Graphical Abstract:**

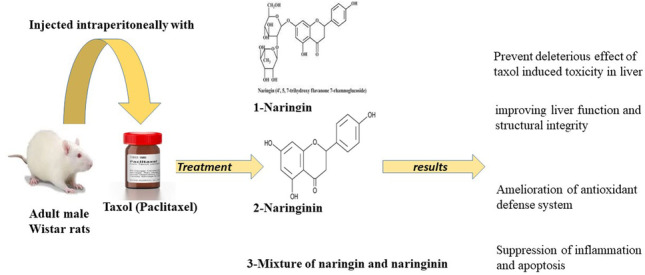

## Introduction

Taxol (paclitaxel) is an essential member of the taxane family and is regarded as an effective anti-tumoral agent (Zhu and Chen 2019). Taxol is one of the most effective natural anticancer medications that have been discovered (Yu et al. 2022). Paclitaxel (trade name Taxol®), a lipophilic chemical produced from *Taxus brevifolia*, can suppress cellular division, motility, activation, secretory activities, and signal transduction (Roberts et al. 1982; Belotti et al. 1996; Jackson et al. 1997; Hui et al. 1998; Wang et al. 1998; Giannakakou et al. 2001). Numerous studies have demonstrated the efficacy of Taxol treatment as a supportive therapy for cancers such as ovarian cancer (Gu et al. 2019), breast cancer (Hou et al. 2019; Tomko et al. 2019), and nasopharyngeal carcinoma (Gao et al. 2018; Wang et al. 2020). Taxol resistance, a challenging issue for clinical therapy and long-term anticancer effects (Song et al. 2015; Gao et al. 2018; Tomko et al. 2019; Wang et al. 2020), however, caused the overall efficacy to remain unsatisfactory. Because of its poor solubility in water and poor tolerance of the excipients used in the formulation, Taxol has a restricted therapeutic applicability (Ezrahi et al. 2019). Taxol is processed in the liver and eliminated in the bile like other cytostatic agents (Horwitz et al. 1993; Manzano et al. 1996; Stenina 2003; Vaclavikova et al. 2004). Side effects of Taxol treatment include elevated liver biomarkers and histopathological damage in the Taxol group, a confirmation of its hepatotoxic effect [(Xie et al. 2015; Adikwu et al. 2019; Gür et al. 2022). Generally, chemotherapy-induced liver toxicity is believed to be secondary to reactive oxygen species (ROS) generation and is proposed to induce tumor cell apoptosis (Lim et al. 2010). Taxol was also reported to cause organs’ oxidative stress and toxicity (Cristiano et al. 2022; Ali et al. 2023). Therefore, current research is focused on increasing Taxol’s bioavailability (Gade et al. 2022).

Medicinal plants and their bioactive compounds have anti-angiogenic, antioxidant, sedative, and analgesic properties, and the beneficial effects registered to date promote their usage in endometriosis management (Ashrafizaveh et al. 2019). Flavonoids are a vast group of phenolic chemicals found widely in plants and are key components in many traditional treatments (Sun et al. 2013). Additionally, flavonoids are secondary plant metabolites that contribute to flower color and aroma, cellular growth regulation, pollinator insect attraction, and biotic and abiotic stress protection (Rodríguez De Luna et al. 2020). Moreover, flavonoids are best known for their antioxidant effect, enzyme regulation, anti-inflammatory, vasculoprotection, and anti-diabetic effects (Tungmunnithum et al. 2018; Dias et al. 2021).

Naringin and its aglycone, naringenin, are the principal flavonoids found in grapefruit (Sayre et al. 2012) and have a variety of pharmacological potentialities, such as antioxidant, anti-free radical, and anti-lipoperoxidation (Gerçek et al. 2021). Naringin and naringenin were reported to act as antitumor compounds by suppressing carcinogenesis through several mechanisms and could be hopeful candidates for new safe anticancer therapies following further research (Memariani et al. 2020).

Due to the common use of Taxol in the chemotherapy of many different forms of tumors and its adverse hepatotoxic effects, the goal of this work was to examine the potential preventive benefits of naringin and naringenin on hepatotoxicity induced by Taxol and elucidate their roles in tailoring oxidative stress, inflammation, and apoptosis.

## Materials and methods

### Experimental animals

In this study, fifty mature male Wistar rats weighing 130–150 g served as the test subjects. They were purchased from the Vacsera Vaccination Centers’ Animal Facilities in Helwan, Cairo, Egypt. The animals were housed in polypropylene cages with stainless steel covers that were well ventilated, at a typical air temperature of 25 ± 5 °C, and with 12-h cycles of light and darkness every day. Before the experiment began, the animals were overseen for 15 days to rule out any concurrent infections. Animals were given unlimited access to water and a daily, balanced standard food. All animal processes followed the recommendations and guidance of institutional animal care and use committee (IACUC), Faculty of Science, Beni-Suef University, Egypt (Ethical Approval Number: BSU/FS/2017/7). All efforts have been done to alleviate the animals’ pain, distress, and discomfort.

### Chemicals

Taxol (paclitaxel) of batch number: 7E05628 was purchased from Bristol-Myers Squibb global biopharmaceutical company. Sigma (MO, USA) provided naringenin and naringin (batch codes: BCBM4171V and BCBJ2179V, respectively). Alkaline phosphatase (ALP) reagent kits, alanine transaminase (ALT), aspartate transaminase (AST), gamma-glutamyltransferase (GGT), and GGT were all bought from Biosystem S.A. (Spain) with catalogue numbers of M11533c-21, M11531c-21, M11584c-11, and M11592-0610, respectively. Lactate dehydrogenase (LDH) reagent kit (catalogue number: MX41214) acquired from Spin React (Spain). Albumin and total bilirubin kits (catalogue numbers 10560 and 10,742 respectively) were attained from HUMAN Gesellschaft für Biochemica und Diagnostica mbH, Wiesbaden, Germany. Verso 1-Step RT-PCR Reddy Mix Kit from Thermo Scientific (Applied Biosystems, Foster City, CA, USA) (catalogue number: AB-1454-LD). All other chemicals were purchased commercially and were of analytical quality.

### Experimental design

**Fig. 1:**
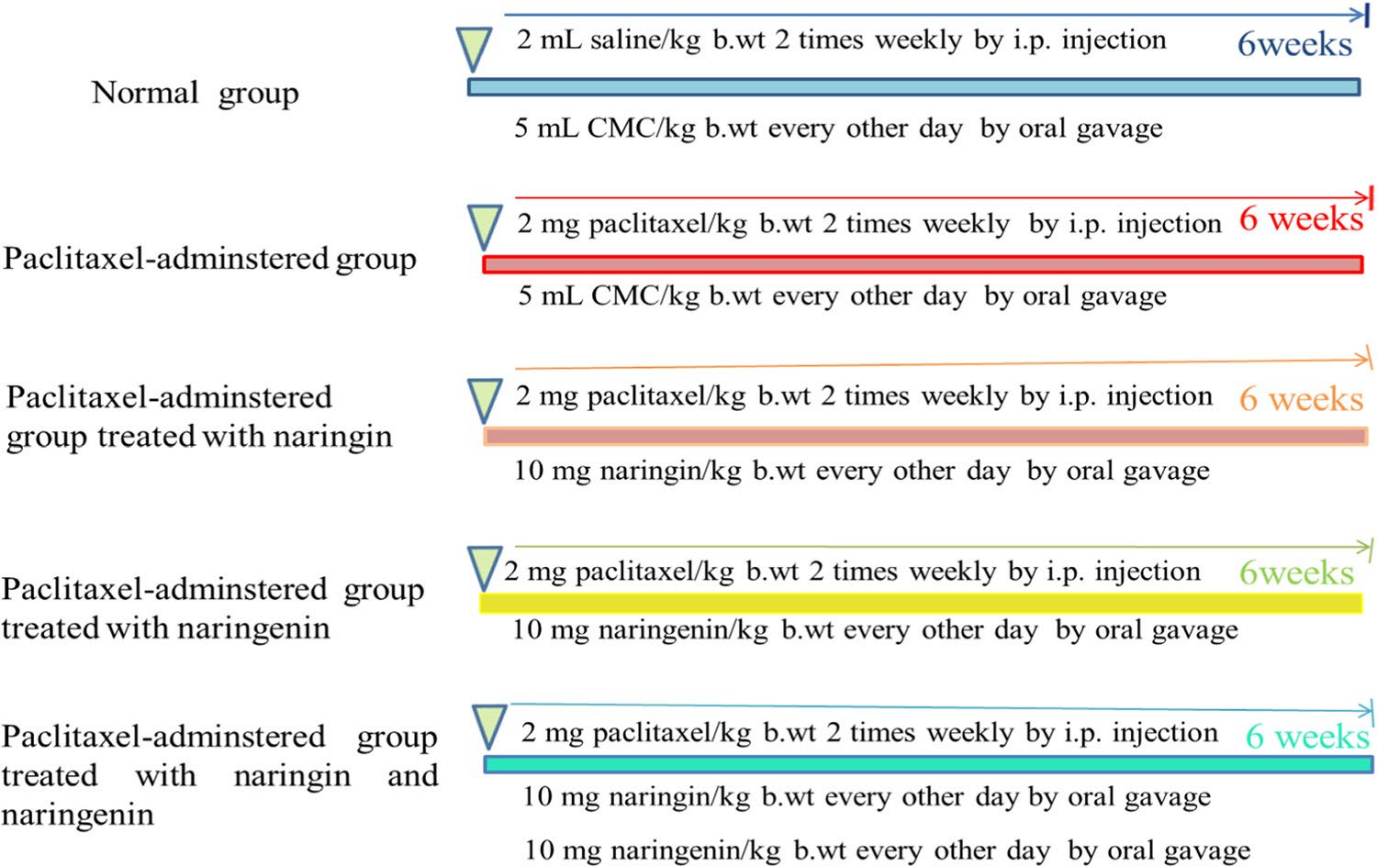
Animal grouping and experimental design

The adult male Wistar rats used in this study were divided into five groups, each consisting of ten rats (Fig. 1):

1. Normal group: For 6 weeks, rats in this group received 2 ml of saline intraperitoneally twice a week and 5 ml of 1% carboxy methylcellulose (CMC)/kg body weight (b.wt) orally every other day.
2. Taxol-administered control group: For 6 weeks, rats in this group received intraperitoneal (i.p.) Taxol at a dose level of 2 mg/kg b.wt (Gao et al. 2016) twice a week, along with oral administration of the equal volume of 1% CMC (5 ml/kg b.wt) every other day.
3. Taxol-administered group treated with naringin: Similar to the Taxol-administered control group, this group of rats received i.p. Taxol before receiving naringin orally every other day for 6 weeks at a dose of 10 mg/kg body weight (Reddy et al. 2008) (dissolved in 5 ml 1% CMC).
4. Taxol-administered group treated with naringenin: Similar to the Taxol-administered control group, this group of rats received i.p. Taxol before receiving naringenin orally every other day for 6 weeks at a dose of 10 mg/kg body weight (Fallahi et al. 2012) (dissolved in 5 ml 1% CMC).
5. Taxol-administered group treated with mixture of naringin and naringenin: Similar to the Taxol-administered control group, rats in this group received i.p. Taxol before receiving a combination of naringin and naringenin orally every other day for 6 weeks at a dose of 10 mg/kg b.wt (dissolved in 5 ml 1% CMC).

### Blood and tissue sampling

Blood was drawn from the jugular vein of each animal at the conclusion of the experiment, placed in gel and clot activator tubes, and then centrifuged at 3000 rpm for 15 min. For various biochemical analyses, the supernatant sera were quickly collected, divided into four servings for each animal, and kept at − 30 °C. Livers were quickly excised after decapitation and dissection. For histological examinations, each animal’s liver portions (5 mm^3^) were settled in 10% neutral buffer formalin. Each rat’s liver pieces (0.5 g) were homogenized in 5 mL 0.9% NaCl. The homogenate was centrifuged at 3000 rpm for 15 min to separate the supernatants, which were then frozen at 30 °C until they were used to detect markers for oxidative stress and the antioxidant defense system. Before being employed for RNA isolation and reverse transcriptase-polymerase chain reaction (RT-PCR) analysis, liver Sects. (3 mm^3^) were stored in sterile Eppendorf tubes at − 70 °C.

### Biochemical investigation of liver function

The activities of serum ALT and AST were measured following the method of Gella et al. (1985). Serum activities of GGT and ALP were analyzed following the techniques of Schumann et al. (2002) and Schumann et al. (2011), respectively. The activity of LDH was measured following the method of Young (2000). Albumin and total bilirubin levels were measured following Doumas et al. (1971) and Jendrassik and Grof (1938), respectively.

### Assessment of oxidative stress and antioxidant status in the liver

Liver LPO (lipid peroxidation) was estimated following the technique of Preuss et al. (1998). Briefly, to precipitate the protein, 75 μl of 76% (TCA) trichloroacetic acid was added to 0.5 ml liver homogenate. The separated supernatant was then given 175 μl of 1.07% TBA (thiobarbituric acid) as a color-developing agent. After 30 min in a water bath at 80 °C, the formed weak pink color was detected at 532 nm. MDA (malondialdehyde) was used as standard. By adding 0.5 ml of 5,5′-dithiobis (2-nitrobenzoic acid), Ellman’s reagent (as a color-developing agent), and phosphate buffer solution (pH 7), to the homogenate supernatant following protein precipitation, liver GSH (glutathione) content was estimated using the method of Beutler et al. (1963) with some modifications. The generated yellow colors in samples and GSH standards were contrasted with a blank at 412 nm.

Following the methodology of Matkovics et al. (1998), which is based on the GSH detection that is converted to GSSG (oxidized glutathione) by the enzyme through the residual GSH detection and subtraction from the total, liver GPx (glutathione peroxidase) activity was measured. Shortly after, 350 µL of Tris buffer (pH 7.6), 50 µL of GSH solution (2 mM), and 50 µL of H_2_O_2_ (3.38 mM) were added to a Wasserman tube. After 10 min of incubation, the remaining GSH content was measured at 430 nm using the previously described technique for GSH measurement. The blank test was carried out by substituting 100 µL of distilled water for 50 µL of samples and 50 µL of GSH solution, while the standard test was carried out by substituting 50 µL of distilled water for 50 µL of samples. The enzyme activity can then be determined once the sample’s residual GSH content has been identified and the GSH has been converted to the oxidized form (GSSG).

Using Marklund and Marklund’s approach, liver SOD (superoxide dismutase) activity was assessed Marklund and Marklund (1974). Pyrogallol’s autooxidation is inhibited by SOD in the process, which is the basis of the method. It is necessary for the procedure to have superoxide ions present. The amount of enzyme that inhibits extinction changes by 50% in a minute is considered to be one unit of enzyme when compared to the control.

### Histopathological examination

Each rat was sacrificed, decapitated, and dissected, and the liver was immediately removed and injected with saline. Different groupings of rat liver fragments were collected, preserved for 24 h in 10% neutral buffer formalin. Following a thorough rinse in tap water, the samples were dehydrated using a series of ethyl alcohol dilutions (50%, 70%, 90%, 95%, and 100%). In a furnace set to 56 °C for 24 h, samples were cleaned with xylene before being submerged in paraffin wax. Sections of 4 micron thickness were made from paraffin wax tissue blocks with a slide microtome. For a standard examination, the tissue sections were collected on glass slides, dewaxed, and stained with hematoxylin and eosin (H&E) stain. The examination was carried out using a light electric microscope (Banchroft et al. 1996).

### Gene expression analysis

To determine the mRNA expression of alpha-fetoprotein (AFP), total RNA was isolated from the liver tissue using the methods of Chomzynski and Sacchi (1987) and Hassan et al. (2021) and Thermo Scientific Verso 1-Step RT-PCR Reddy Mix Kit (Applied Biosystems, Foster City, CA, USA) as directed by the manufacturer in the presence of particular primers. The forward and reverse primer sequences for AFP are 5′dAACAGGGCGATGTCCATAA3′ and 5′dAATGGTGGGAGCATACAGG3′, respectively (Tsamandas et al. 2007). The AFP data was expressed as a fold change from the typical control.

### Immunohistochemical detection of caspase-3

The Department of Pathology at the National Cancer Institute processed, blocked, and sectioned the liver samples into 5-µm-thick sections before mounting them on positively charged slides (Fisher Scientific, Pittsburgh, PA) after the liver samples had been fixed in 10% neutral buffered formalin. To process the caspase-3 reactivity, Galaly et al. (2014) and Ahmed and Ahmed (2014) procedures were employed. Briefly, liver sections were exposed to diluted primary caspase-3 antibody (Santa Cruz Biotechnology, Santa Cruz, CA, USA) for 1 h following antigen retrieval. Biotinylated secondary antibody (DakoCytomation Kit) was diluted and incubated for 15 min at 37 °C. Horseradish peroxidase-conjugated streptavidin (DakoCytomation Kit) was then added, and a further 15 min were added for incubation. The bound antibody complex was discovered through the reaction of the 3, 3′-diaminobenzidine (DAB) substrate and counterstaining with hematoxylin. Since all liver sections were treated simultaneously under same conditions using identical antibody dilutions, the immune staining was comparable between research groups. Each preparation underwent a negative control (a slide without a primary antibody). To assess the level of cell immunological positive, the slides were inspected under a light microscope. Images of liver sections were captured using a digital camera (Leica, DM2500M Leica, Wetzlar, Germany). The labeling was examined and analyzed using the free software ImageJ (1.51d) (Varghese et al. 2014). ImageJ was used to calculate the integrated intensities (in pixels) of caspase-3’s favorable response.

### Statistical analysis

For statistical analysis, IBM software’s SPSS computer program (version 22), for social sciences, was employed. A one-way analysis of variance (ANOVA) test was used to establish the significance of group means, and then a Tukey’s post hoc test was used to compare mean values pairwise. The results were represented as means ± standard errors of means, and the differences were deemed significant at *p* < 0.05.

## Results

### Effect of treatment on serum biomarkers of liver function

**Fig. 2:**
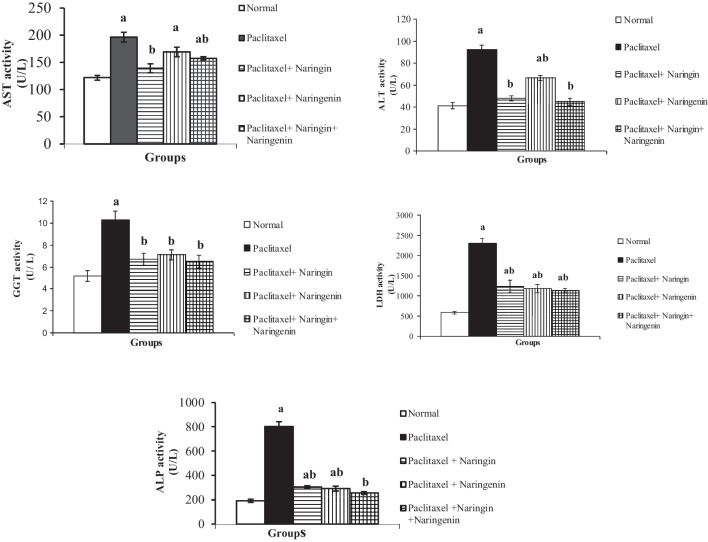
Activities of serum enzymes related to liver function in normal, Taxol-administered control and Taxol-administered groups treated with naringin, naringenin, and their combination. Results are expressed as mean ± SEM of *n* = 6. ^a^*p* ˂ 0.05: significant in comparison with the normal group. ^b^*p* ˂ 0.05: significant in comparison the Taxol-administered group

**Fig. 3:**
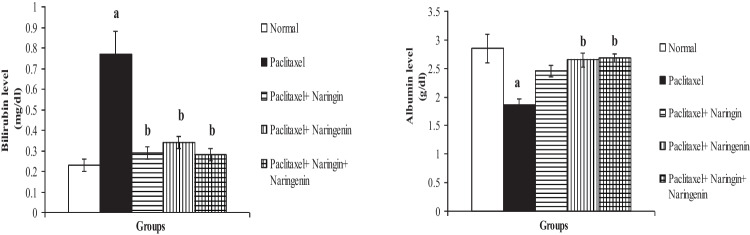
Serum bilirubin and albumin levels in normal, Taxol-administered control and Taxol-administered groups treated with naringin, naringenin, and their combination. Results are expressed as mean ± SEM of *n* = 6. ^a^*p* ˂ 0.05: significant in comparison with the normal group. ^b^*p* ˂ 0.05: significant in comparison the Taxol-administered group

The serum AST, ALT, GGT, LDH, and ALP activities as well as the total bilirubin level were considerably (*p* < 0.05) elevated in the rats after receiving intravenous Taxol for 6 weeks. In contrast, the treatment of Taxol caused a substantially lower (*p* < 0.05) serum albumin level with a drop in percentage of 34.39% when compared to the normal group. When compared to Taxol-administered rats, naringin, naringenin, and their combination significantly improved the serum AST, ALT, GGT, LDH, and ALP activities, as well as bilirubin concentration and albumin level (Figs. 2 and 3).

### Effects of oxidative stress on the liver and antioxidant defense system parameters

**Fig. 4:**
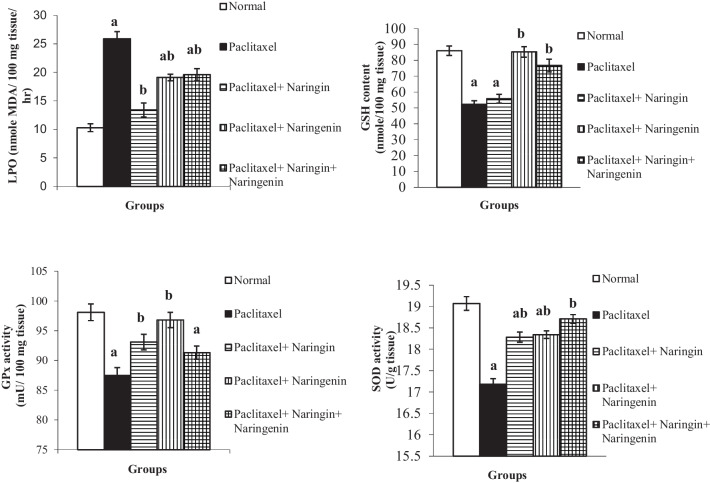
Liver LPO, GSH content, and SOD and GPx activities in normal, Taxol-administered control and Taxol-administered groups treated with naringin, naringenin, and their combination. Results are expressed as mean ± SEM of *n* = 6. ^a^*p* ˂ 0.05: significant in comparison with the normal group. ^b^*p* ˂ 0.05: significant in comparison the Taxol-administered group

When Taxol was administered to normal rats, as shown in Fig. 4, it considerably (*p* < 0.05) boosted liver LPO (+ 151.46%) while significantly (*p* < 0.05) lowering GSH level, SOD, and GPx activities in comparison to normal control rats. Naringin and naringenin, either singly or in combination, were administered orally, and the LPO was improved substantially, falling by 48.26, 26.25, and 24.32%, respectively. Additionally, liver GSH content was markedly increased due to the treatment with naringin, naringenin, and their mixture with percentage changes of + 6.60, + 53.07, and + 37.61% respectively. The naringenin treatment and its mixture with naringin resulted in a considerable increase (*p* ˂ 0.05) in GSH content, whereas the naringin treatment produced a non-significant increase (*p* > 0.05). Furthermore, the three treatments ameliorated the activities of liver SOD recording significant (*p* ˂ 0.05) percentage changes of + 6.41, + 6.73, and + 8.91% respectively and GPx which recorded significant (*p* ˂ 0.05) changes of + 6.4 and + 10.63% for naringin and naringenin respectively while their mixture induced a non-significant rise (*p* > 0.05) in GPx activity with change of + 4.34%.

### Liver histopathological Effects

**Fig. 5:**
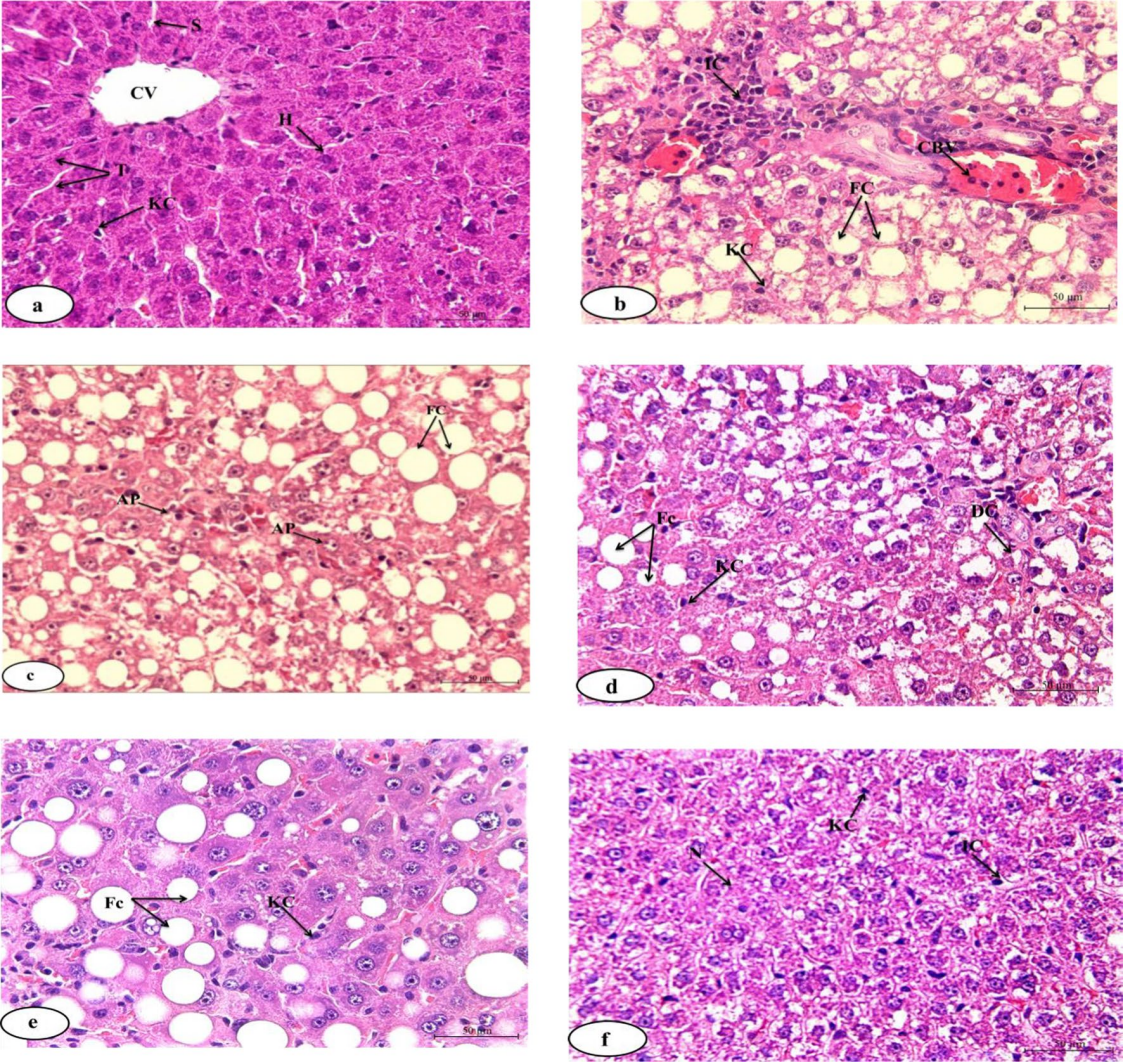
Photomicrographs of liver sections of different experimental groups: **a** photomicrograph of liver section of normal rats, demonstrating the normal histological structure of a hepatic lobule, hepatocytes (H), trabeculae (T), sinusoids (S), central vein (CV), Kupffer cells (KC); **b**, **c** Photomicrographs of liver section of Taxol-administered rats, indicating congested blood vessels (CBV) in the portal area, severe fatty change (FC) and inflammatory cell infiltration (IC) in the portal area, sever proliferation of Van Kupffer cells (KC), and apoptotic cells (AP); **d** photomicrograph of liver section of Taxol-administered rats treated with naringin indicating moderate degenerative changes (DC), fatty change (FC), and moderate proliferation of Van Kupffer cell (KC); **e** photomicrograph of liver section of Taxol-administered rats treated with naringenin indicating moderate fatty change (FC), inflammatory cells (IC), and moderate proliferation of Kupffer cells (KC); and **f** photomicrograph of liver section of Taxol-administered rats treated with mixture of naringin and naringenin showed moderate necrosis (N), minimal inflammatory cells (IC), and mild proliferation of Van Kupffer cells (KC). (H&E; 400 ×)

Various histopathological lesions in liver specimens of experimental groups are illustrated in Table 1 and Fig. 5. The histological investigation from normal control rats’ liver sections revealed normal histological architecture (Fig. 5a). However, severe to moderate pathological hepatic lesions were found in the Taxol-administered group including marked congestion in the central veins, blood vessels of the portal area, severe fatty changes, and moderate necrotic changes. Meanwhile, pyknotic changes in some hepatocytes, moderate mononuclear leucocytic infiltration, Kupffer cell proliferation, and marked apoptosis were seen (Fig. 5b and c). Improvements in these changes were found in the Taxol-administered groups treated with naringin and/or naringenin. Mild fatty changes, necrotic changes, inflammatory cells, necrosis, degenerative changes, Kupffer cell proliferation and apoptosis, and minimal congestion were observed in Taxol-administered groups treated with naringin (Table1; Fig. 5d). Moderate fatty changes and mild inflammatory cells, degenerative changes, and Kupffer cell activation as well as minimal necrosis, congestion and apoptosis were detected in the Taxol/naringenin-treated group (Table 1; Fig. 5e). Rats treated with the mixture elucidated quite improvement of the hepatic histological changes compared to other treated rats. Mild fatty and degenerative changes were noticed while other lesions exhibited minimal scores (Table 1; Fig. 5f).

**Table 1:** Pathological hepatic lesion scores in the tested groups

Lesion groups	Fatty changes	Necrotic changes	Inflammatory cells	Degenerative changes	Congestions	Kupffer cell proliferation	Apoptosis
Normal	0	0	0	0	0	0	0
Taxol	IV	III	III	III	III	IV	III
Taxol + naringin	II	II	II	II	I	II	II
Taxol + naringenin	III	I	II	II	I	II	I
Taxol + naringin + naringenin	II	I	I	II	I	I	I

Histopathological lesion scores are categorized 0: absence, I: minimal, II: mild, III: moderate, and IV: severe

### Effects of treatment on liver tissue mRNA expression of AFP

**Fig. 6:**
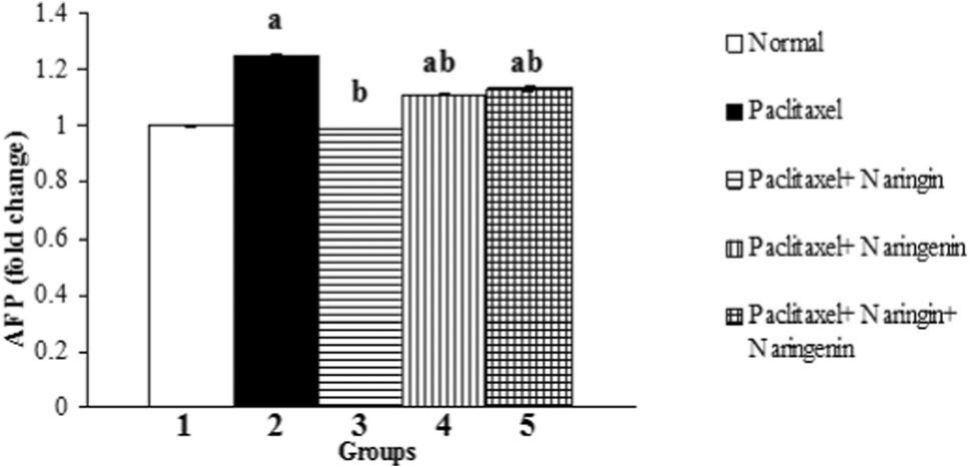
The mRNA expression of AFP in normal, Taxol-administered control, and Taxol-administered groups treated with naringin, naringenin, and their combination. ^a^*p* ˂ 0.05: significance compared with normal control. ^b^*p* ˂ 0.05: significance compared with Taxol-administered rats

The administration of Taxol to rats for 6 weeks induced a significant (*p* ˂ 0.05) elevation in mRNA expression of AFP. Naringin, naringenin, and their mixture in Taxol-administered rats induced significant decreases (*p* ˂ 0.05) in the elevated mRNA expression of AFP (Fig. 6).

### Effects of treatment on liver tissue immunohistochemistry

**Fig. 7:**
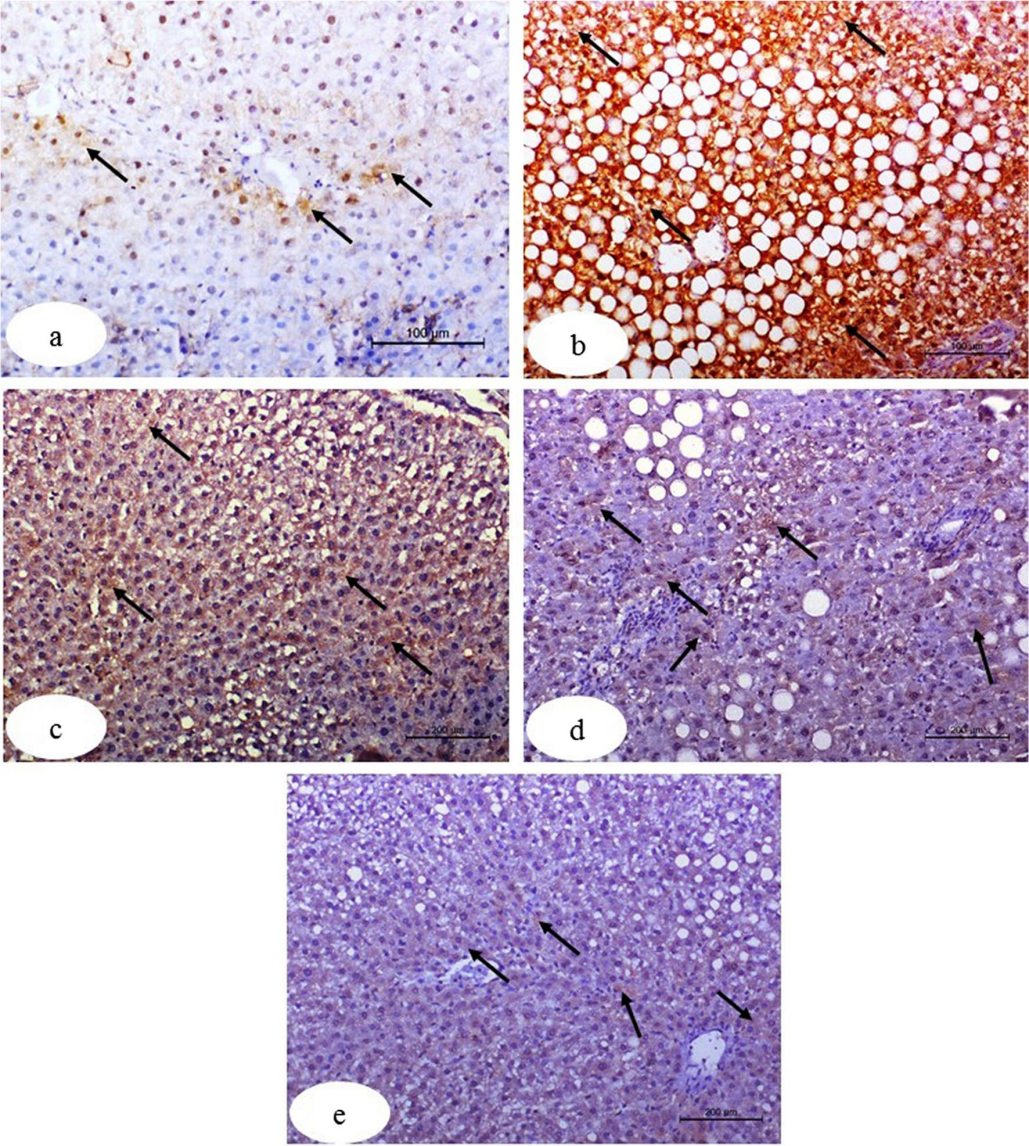
Photomicrographs of immunohistochemically stained liver sections for the detection of caspase-3 showing very weak expression in the normal group (**a**), very strong expression in the group that received Taxol (**b**), moderate expression in the group that received Taxol and was treated with naringin (**c**), mild expression in the group that received Taxol and was treated with naringenin (**d**), and weak expression in the group that received Taxol and was treated with both naringin and naringenin (**e**)

Table 2 and Fig. 7 show the immune-histochemical reactivity of caspase-3. When Taxol was given to rats for 6 weeks, the caspase-3 activity was considerably (*p* < 0.05) higher than it was in the control group. In Taxol-treated rats, naringin, naringenin, and their combination all significantly reduced the enhanced caspase-3 activity (*p* < 0.05), with the combination of naringin and naringenin being the most potent.

**Table 2:** Liver caspase-3 protein expression in normal, Taxol-administered control, and Taxol-administered groups treated with naringin, naringenin, and their combination

Parameter groups	Caspase-3 (area %)	% change
Normal	6.924 ± 0.09	**–**
Taxol	37.630 ± 1.09^a^	4.44%
Taxol + naringin	21.054 ± 1.17^ab^	− 0.44%
Taxol + naringenin	15.285 ± 1.03^ab^	− 0.59%
Taxol + naringin + naringenin	9.782 ± 0.06^b^	− 0.74%

Results are expressed as mean ± SEM of *n* = 6. Percentage changes were calculated by comparing the Taxol-administered group with the normal, and the Taxol-administered groups supplemented with naringin and naringenin (either singly or in combination) with the Taxol-administered control

^a^*p* ˂ 0.05: significant in comparison with the normal group

^b^*p* ˂ 0.05: significant in comparison the Taxol-administered group

$$\%\;\mathrm{change}=\left(\frac{Final\;value-Initial\;value}{Initial\;value}\right)\times100\;\left(\mathrm{Zaazaa},\;2022\right)$$

## Discussion

Taxol was known to treat numerous malignancies, especially aggressive and metastatic breast cancer, ovarian cancers, lung cancers, pancreatic cancer, and many other malignancies (Klein and Lehmann 2021). However, Taxol has induced non-specific reactive hepatitis and elevated hepatic enzymes, as well as liver tissue histopathological damage in rats (Ermolaeva et al. 2008). Additionally, it is reported to cause hypersensitivity, peripheral neuropathy, nephrotoxicity, myelotoxicity and mucositis (Zang et al. 2019).The current study revealed the impact of Taxol-induced liver tissue, and hepatocellular damage was confirmed biochemically by measuring serum activities of cytosolic enzymes (AST, ALT, and LDH), which were significantly elevated. Additionally, membrane-bound enzyme activities (GGT and ALP) and serum bilirubin levels are increased, thereby revealing hepatobiliary obstructions. These findings are in line with the previous research by Costa et al. (2018) who found that Taxol induces hepatic toxicity by increasing AST, ALP, GGT, and bilirubin. High liver enzyme levels are indicative of cellular leakage and hepatocyte membrane damage (Choudhary and Devi 2014). Albumin is systematically included among the parameters used in the nutritional evaluation and has recently become more widespread (Numeroso et al. 2008). Albumin deficiency indicates various liver disorders (Carvalho and Machado 2018). The present study revealed that Taxol-administered rats exhibited significantly decreased serum albumin levels in concordance with the study of Park et al. (2012).

Histopathological liver tissue investigation in the Taxol-intoxicated rats supported these biochemical results. The liver exhibited congested central vein, moderate necrosis, moderate inflammatory cells, and severe Kupffer cell proliferation. The present results are following the findings of Król (1998) who observed that Taxol causes histopathological liver damage. Furthermore, Karaduman et al. (2010) indicated heightened and time-dependent liver tissue degeneration and necrosis in mice in the Taxol group.

The previous deleterious biochemical and histological alterations were associated with a marked liver LPO elevation and decreased non-enzymatic antioxidant (GSH) content and enzymatic antioxidant (SOD and GPx) enzyme activities. Such findings are consistent with those of a number of other researchers (Alexandre et al. 2007; Hadzic et al. 2010; Panis et al. 2012) who indicated that Taxol treatment results in a lower antioxidant status. Moreover, Taxol exerts cytotoxic effects by generating ROS and causing oxidative stress (Meshkini and Yazdanparast 2012; Vera-Ramirez et al. 2012; Ilinskaya et al. 2015). Commonly, anti-cancer chemotherapeutic drugs attenuate antioxidant defense system and evoke the oxidative stress and production of damaging free radicals to the liver and other organs (Ahmed et al. 2019a, b; Mahmoud et al. 2020, 2021; Ahmed et al. 2022a, 2022b).

AFP is a translation product of the albuminoid gene family and is characterized as an embryo-specific glycoprotein that is associated with a tumor (Beattie and Dugaiczyk 1982; Murray and Nicholson 2011). Failure of AFP decline is usually seen as a sign of chemoresistance or existing residual tumors, requiring further treatment and predicting an unfavorable prognosis (Dällenbach et al. 2006; de la Motte Rouge et al. 2016). Taxol administration in the current research caused mRNA overexpression of tumor marker AFP. These findings agree with those of Takeyama et al. (2007) who showed several new hepatic metastases and increased serum AFP within 5 months in a case report of a patient with multiple hepatic metastases receiving combined chemotherapy, including TS-1 and Taxol.

Immunohistochemical results of the liver section in the Taxol group showed a significantly increased caspase-3 activity. Thus, Taxol is suggested to activate apoptosis in Wistar rats. These findings agree with Gu et al. (2017) who considered Taxol to induce apoptosis and elevate caspase-3 activity. Additionally, Lu et al. (2005) showed that Taxol causes apoptosis through caspase-3 activation in human osteogenic sarcoma cells.

The combined use of Taxol with other traditional medicines was used to improve susceptibility to Taxol while minimizing its dose (Chen et al. 2012; Lee et al. 2014). Flavonoids have been numerically shown to suppress tumor cell growth in vitro and in vivo (Turati et al. 2015). In this investigation, the oral administration of naringin and/or naringenin resulted in a reduction in serum ALT, AST, ALP, GGT, and LDH activity, along with total bilirubin levels, while increasing serum albumin levels. Improvements in biochemical parameters related to liver function were linked to improvements in liver histological architecture. Meanwhile, naringin and/or naringenin decreased liver LPO and heightened GSH content, SOD, and GPx activities after 6 weeks of treatment. These findings go hand in hand with the previous ones of Ahmed et al. (2019a, b) who observed that naringin and naringenin amended changes caused by acetaminophen in liver enzyme (ALT, AST, ALP, GGT and LDH) activities, as well as total bilirubin level, liver LPO, and antioxidant parameters and also attenuate the damage of hepatic tissues.

Additionally, El-Mihi et al. (2017) found that naringin has a protective and therapeutic effect against thioacetamide-induced liver injury and fibrogenesis. Moreover, different doses of naringin were determined protective and effective for cyclophosphamide-induced hepatotoxicity (Bülbül et al. 2018). Additionally, naringenin inhibited the elevation of ALT, AST, and bilirubin in lambda-cyhalothrin-treated rats (El-Saad and Abdel-Wahab 2020). Moreover, Malayeri et al. (2022) found that naringenin enhanced methotrexate-induced alterations in the activities of AST, ALT, and ALP in the liver of rats. Moreover, Cavia-Saiz et al. (2010) mentioned that naringin and naringenin are powerful free radical collectors and inhibit LPO.

Moreover, naringin and naringenin treatment in Taxol-administered rats suppressed the expression of (AFP and caspase-3). The treatment with their mixture was effective in decreasing AFP and caspase-3. The antioxidant characteristics of naringin and naringenin, as well as their ability to scavenge free radicals, may explain their inhibitory effects on Taxol-induced apoptosis and inflammation. Previous publications backed up this assertion, which indicated that both naringin and naringenin have a strong anti-free radical and antioxidant action (Renugadevi and Prabu 2009; Sahu et al. 2014; Alam et al. 2014). Additionally, naringin has antioxidant, anti-inflammatory, and DNA-protecting properties (Gelen et al. 2018). As mentioned also, naringenin can alleviate inflammation and cell death (Jayaraman et al. 2012; Lou et al. 2012; Wali et al. 2020).

## Conclusions

The administration of naringin, naringenin, and their mixture potentially prevented the deleterious effects of Taxol-induced toxicity in the liver. The mixture of naringin and naringenin was found to be the most potent in improving liver function and structural integrity. The ameliorative effects of naringin and/or naringenin could be mediated via antioxidant defense system enhancement as well as inflammation and apoptosis suppression. More experimental and clinical studies are needed, however, to determine the molecular mechanisms of action by checking signaling pathways involved in oxidative stress, antioxidant defense, inflammation and apoptosis and to assess the efficacy and safety of naringin and/or naringenin in humans.

## Data Availability

All data are contained in the article.
